# Lay Health Promoters Empower Neighbourhoods-Results From a Community-Based Research Programme in Southern Sweden

**DOI:** 10.3389/fpubh.2022.703423

**Published:** 2022-04-26

**Authors:** Margareta Rämgård, Helen Avery

**Affiliations:** ^1^Department of Care Science, Malmö University, Malmö, Sweden; ^2^Centre for Advanced Middle Eastern Studies, Lund University, Lund, Sweden; ^3^Department of Languages, Linnaeus University, Växjö, Sweden

**Keywords:** empowerment, community-based participatory research (CBPR), health equity, health promoters, community health work

## Abstract

This article focuses on the role of lay health promoters in the Lindängen initiative, a community-based participatory research (CBPR) programme for health promotion that started 2016 in a low-income neighbourhood in the outskirts of Malmö, southern Sweden. The programme aimed to support equitable health and develop an innovative model for community health promotion. The purpose of this article is to describe the role of the lay health promoters in the initiative and discuss the challenges of their position mediating between residents of the neighbourhood and the established institutions, using interviews, meeting notes and focus groups sessions with the health promoters and local stakeholders. Lay health promoters' role and function developed in a collaborative process of networking with local stakeholders and citizens in local meeting places. Their work was based on their credibility in their role, in combination with the use of a CBPR model that was open to innovation, with a strong focus on actively engaging members of the community. This allowed the lay health promoters to take political and social stances towards various issues, and to start to concretely address the social determinants of health in the community, as well as express recommendations to policymakers. Based on these experiences, the lay health promoters gained clearer insights into the institutional and structural conditions that impact their community. The position they had in the process of the programme resulted in empowerment and a new local association for health promotion; LindängenKraft (LindängenPower) driven by the health promotors and community members.

## Introduction

Health promotion is an approach based on work with communities aiming at preventing disease and improving health and well-being, through empowerment and strengthening capacity within communities. At the core of health promotion, as defined by the World Health Organization in the Jakarta Declaration, lies community empowerment: “*Health promotion is carried out by and with people, not on or to people. It improves both the ability of individuals to take action, and the capacity of groups, organizations or communities to influence the determinants of health”* (1).

This article focuses on the role of lay health promoters (LHPs) in the Lindängen initiative, a community-based participatory and challenge-driven research programme in a low-income neighbourhood in the outskirts of Malmö, southern Sweden. The initiative aimed to support equitable health and develop an innovative model for community health promotion, drawing on the ethos of the Jakarta Declaration and using Wallerstein's Community-Based Participatory Research (CBPR) approach (2, 3). The LHPs in Lindängen were recruited in the neighbourhood and came to play a key role in mobilising the inhabitants and in developing the collaborative structures of the community health promotion.

Sweden differs from many other countries in that the non-governmental not-for-profit sector does not traditionally play an important part in ensuring citizens' welfare concerning health and social care. There is little experience of LHPs, and it is difficult to directly transfer insights from the international literature concerning community health work and health promotion in communities. Although Sweden has a long-established tradition of local associations, for instance for tenants or special interests such as sports, grassroots organisations representing communities as a whole are rare. Social work and neighbourhood development initiated by authorities faces difficulties in bridging differences in perspectives (4–7), and citizen engagement tends to be limited (8). This article therefore also discusses the difficulties in connecting community-based health promotion involving LHPs with the Swedish institutional structures, and some of the perceived challenges in ensuring the duration of the initiative beyond the period of initial funding. A brief description of the structure and processes of the programme is provided as a background.

## Materials and Methods

### Purpose

The purpose of this article is to summarise key aspects of the role of the lay health promoters (LHPs) in the Lindängen initiative for equitable health, and to discuss the main challenges of their position as mediators between residents of the neighbourhood and the established institutions.

### Study Context: The Lindängen Programme for Equal Health

Sweden has a high average life expectancy but, like many other countries, faces challenges with health inequity. Inequalities are particularly noticeable in urban neighbourhoods of the country's major cities (9). In 2013, the Malmö Commission—a city-level initiative inspired by the WHO, and composed of Malmö City, local stakeholders and academia—conducted an investigation of the situation in Malmö. The Commission concluded with a recommendation to develop health promoting interventions using a multisectoral approach, which would take into consideration local needs, through actively involving communities, in an attempt to reduce health inequity among Malmö residents (10).

The Lindängen neighbourhood consists of housing estates mainly constructed in the 1970s. Eighty per cent of the 7800 residents are first- and second-generation immigrants. Unemployment rates in the neighbourhood are approximately 45%, compared to an average of 15% for the city as a whole, while the number of ill health days in 2020 per inhabitant ages 20–64 was 50% higher than the city average (11). The Collaborative Innovations for Health Promotion programme had the primary aim of reducing health inequalities in the Lindängen neighbourhood and researching empowerment processes throughout the programme. An additional aim of the programme was to develop a participatory locally grounded model that could be used in other contexts. Besides community members, the model involved academic, public sector, commercial, and not-for-profit actors. Residents were actively involved in the planning phase, defining both objectives and structures. For the period April 2017-August 2019, the Swedish national innovation agency Vinnova contributed with half the funding (2017-01272) while the other half was supplied by 14 organisations, public actors, and companies. The academic partner had earlier, together with the city and certain of the other actors, applied for a smaller Vinnova initiation grant (2016-00421). This funding was in the period April to December 2016 used to prepare the programme. Future workshops and other preparatory activities of the 2016 pre-study involved only the academic research team, the neighbourhood residents, and local actors in the neighbourhood.

The long-term goals of the Lindängen programme were to strengthen processes that foster more equal health, and establish a health-promotional structure with an innovative platform in which the role of LHPs would be central. The function of local “brokers” (12) working as LHPs was thus outlined in the initial funding application. The long-term goals also included identifying a new health promotion model for socially deprived areas. Participation in the programme involved community members in all processes. The health promotion approach should follow CBPR principles and lead to measurable improvements in health. In this framework, the role of the LHPs would be to integrate knowledge from the real world into the “living labs” (13), mobilise local knowledge on empowerment processes, and involve the local community in research and challenge-driven innovation work.

### Participatory Research Approach and Processes

The Lindängen programme adopted a consistent community-based participatory research (CBPR) approach (2, 3) and both structure and content were defined by the community. There was a long process together with the community before activities in the labs could start. For the academic participants, from April 2016 onwards, this firstly involved building trust (14) by spending time in the community at the social meeting places, presenting ourselves and speaking with residents to learn about the context. From these initial contacts, it appeared that activities in the neighbourhood were not driven by residents, although there had earlier existed a grassroots organisation called Street Power (gatukraft). Knowledge about health promotion and perceived needs was lacking, and residents expressed a mistrust in the health care system (3, 13). However, residents also expressed a sense of belonging and “place identity” [see Rämgård (15)].

Three large future workshops (16) involving 150 community members were held in the period June-November 2016 (17), to collectively decide on structure and content for health-promoting activities. An open invitation to attend the future workshops was made to all residents in the neighbourhood, as well as to different community groups, such as a local women's network. The workshops were facilitated with the support of a process leader from outside and an Arabic speaking interpreter nurse, with invited citizens from the community. The workshops started with the question: *What do we need to be healthy?* Answers were grouped into themes, then the work continued with the questions: *What can we do in the area? What do we need to promote healthy living here?* Finally, workshop participants were asked to suggest solutions. The workshops resulted in identifying some urgent matters for the community, notably: lack of physical activity, poor oral health, mental health problems, and insecurity in different places. The citizens in the future workshops also indicated that to facilitate the labs they needed mediators and leaders from the community who knew the area, the local culture and the residents. The activities needed to be free of charge and in the daytime.

After identifying the perceived needs in the neighbourhood, the workshop participants, together with local stakeholders, created a community-based participatory research (CBPR) model for planning health promotion activities in the neighbourhood (2, 18). This comprised a local neighbourhood HUB, a steering committee with partners external to the neighbourhood, as well as processes to ensure coordination, evaluation and further development. Representatives from the dialogue groups formed in the future workshops took action together with the local partners and conducted CBPR planning using Wallerstein's model (3), suggesting roles for the partnership and CBPR interventions. Participants further decided to organise six co-creative health-promoting labs on the different themes identified in the workshops and to recruit LHPs from the neighbourhood.

The LHPs were citizens who had taken part in the initial future workshops and who expressed their interest in assisting the research team and being change agents for their community. They did not have any prior experience of health work. From April 2017 onwards, the CBPR process was used to define the role of the LHPs, which continued to evolve in the following years.

The LHPs created health promotion labs from the perspectives that had been discussed in the future workshops. In the labs, all community members in the areas were invited. As decided in the future workshops, everything should be open for all citizens in the area, but the women also wanted a group only for women to be able to do physical activities. All activities were free of charge, and the community members decided together what they needed in the labs. The LHPs facilitated the labs and had contacts with the citizens on WhatsApp between the activities. Other stakeholders that could be relevant to the labs were invited by the citizens and the LHPs.

A process evaluation of partnership processes, power mechanisms, and experiences of activities was conducted every 6 months, to follow up the planning model and further develop activities and structures. Interviews were conducted with partners in the steering committee, the local HUB, the LHPs, and community members in the labs (*n* = 250), according to interview guides following the planning model (17, 19). The model followed an iterative process, where the future workshops and CBPR planning were anticipated to start again after being evaluated every second year (unfortunately this was not possible because of the pandemic, and public health guidelines that precluded large public meetings).

Focus group meetings with the community members were held together with researchers in all labs during the programme. The results from the focus groups were presented in the steering committee, in which stakeholders representing various structural levels (health care, the city, the university, non-governmental organisations, property owners), together with the LHPs representing the community, discussed structural barriers to changes in policy and practice (13).

In the initial phase of the Lindängen programme, the LHPs were involved in building the model. They also developed their own function that created sustainability in the community through the CBPR programme. After being recruited during the large citizen workshops, the LHPs continued to participate in the CBPR planning work, together with participants from the university and other stakeholders. The planning included a process evaluation model that allowed the LHPs to adjust and refine their work continuously. While each lab was run separately by the LHPs and the neighbourhood residents who participated in them, the LHPs had their own coordination and support group, where they shared experiences, reflected on their practice and developed strategies to deal with any challenges that appeared. Throughout the process, the LHPs were members of the core management groups working with collaboration among the various stakeholders, rather than simply implementing interventions as employees in a more peripheral function.

### Management and Support Structure

The present study focuses on the role of the LHPs, and therefore only provides a brief description of the management and support structure for the Lindängen initiative as a whole. Details concerning the programme's management structure, CBPR processes and the individual health promotion labs have been reported in other studies [see (13, 17, 20–23)]. The LHPs occupied a key position in the structure, since they were involved in the steering committee, the local HUB, and the individual labs, as well as having their own internal meetings.

The steering committee comprised partners on the structural level, with directors from organisations representing relevant sectors in society (17). The University Pro Vice-Chancellor chaired the steering committee (see Table 1), while the project leader (first author) organised the steering committee meetings. The role of the steering committee was to follow developments in the programme and discuss barriers and resources in their own organisations, aiming to find strategies for a more community-driven health promotion programme in the neighbourhood and in the city more generally. The local lay health promoters represented the community in the steering committee.

**Table 1:** Overview of management structure (only listing key actors most relevant to the lay health promoters' work).

**Sectors in society**	**Steering committee (academic partner chaired the meetings)**	**Local HUB (lay health promoters chaired the meetings)**
**Public sector**		
Malmö City, Region of Scania (primary care), FINSAM (financial coordination of rehabilitation measures—bridging sectors and ensuring coordination between regional and city levels)	Director of innovation, primary care political group, health care Programme Director for MILSA (refugees in Sweden)	Local employee from social service, culture workers Malmö City
**Non-governmental sector**		
Red Cross, Save the Children, Scania sports associations	National Red Cross Regional directors	Local employees from Red Cross, Save the Children
**Business sector**		
Oral health company, IT company, pharmacy, employment company, property owners	CEOs and marketing directors	Business partners can participate in the HUB as needed
**Academia**		
	Pro Vice-Chancellor (chair), Project leader, Dean of the Faculty of Dental Care, Dean of the Faculty of Health & Society (Malmö University)	Project leader (nurse/midwife/human geographer)
**Community**	Lay health promoters (*n* = 6)	Lay health promoters (*n* = 6)

The local HUB meetings (Table 1) aimed at resolving practical issues, as well as coordinating, continually assessing and further developing the work. HUB meetings took place in the neighbourhood and were chaired by the LHPs. The key actors were local actors in the area (social workers from Malmö City, and local employees from non-governmental organisations such as the Red Cross, Save the Children), the project leader from the academia, and LHPs from the community. The local actors from the HUB supported the work in the labs, but the LHPs had responsibility for these, while PhD students and researchers from the research teams collaborated with the LHPs in the participatory interventions and PAR research. The academic research team worked in the area on a daily basis during the programme and could also offer health expertise from the various health professions they represented. External speakers and experts were called in to provide lectures and information on health-related topics when requested by lab participants or the LHPs. The academic research team has continued to work closely with the LHPs and community members after the end of the programme period with external funding by Vinnova.

The local HUB had meetings regularly every week, writing notes to take action on. The HUB strengthened the relationship between the various local actors, such as social care, culture workers, health care, property owners, non-governmental organisations and the pharmacist. In this network of intersectoral work, the LHPs were important as brokers to bridge knowledge (tacit knowledge and cultural knowledge) between the sectors and the organisations in the society, as well as spreading tacit knowledge from the community to other institutions in the Swedish society. Over time, this networking built capacity in the neighbourhood and established regular collaboration between different groups in the community.

Because of their advocacy roles both in the steering committee and in the local HUB, the LHPs were in the course of the programme trained in PAR methodologies and Freire's dialogues about empowering communities. This training was also intended to support the LHPs in managing power mechanisms, both with respect to the wider governance, policy and service provision structures, and with respect to their own role and responsibility in giving voice to members of the community. The LHPs also had support from a psychologist in dealing with self-reflection and managing conflicts. Story dialogue methods were notably used to manage conflicts. CBPR projects demand continuous collective ethics work [see e.g., (24, 25)] from all participants, with high awareness of the roles and positions of each participant, self-reflection and discussions on how to manage tensions.

Workload and ethical dilemmas were important issues. The LHPs acted as advocates for the community and worked with social determinants of health between the activities in the labs. This was a burden for them, since they lived in the area and had difficulties in creating boundaries between time working on the project and leisure time for their personal lives. The LHPs were happy to give residents advice on how to make contact with health care and authorities, help fill in different forms, or help refugees understand that the Swedish agenda was not a threat, for instance, but the fact that so many in the community needed their help was a challenge.

### Participants

Six LHPs were recruited in connection with the initial future workshops (16) that defined the priorities and content of the Lindängen initiative (17) and worked part-time for the duration of the programme. Five of them were women and one was a man. The uneven gender distribution was determined by the funding agency Vinnova, where a condition for funding was to support female leadership. Ages of the LHPs ranged between 28 and 57, with one in the interval 20-30 (1F), two aged 30-40 (1F, 1M), two aged 40-50 (2F), and one aged 50-60 (1F). The six LHPs involved in the study are all first-generation immigrants differing in gender, age, and ethnic background. Three women who grew up in Iraq, Iran and Lebanon facilitated labs for physical health, oral health/nutrition, and women's health, respectively. They were all unemployed at the start of the project and as they had children in school they did not want to work full-time. Another woman in her thirties, who arrived in Sweden as a child refugee and had earlier experiences as a volunteer at the Red Cross, facilitated labs for a secure environment and social health, engaging children and young adults in the neighbourhood. The one younger male adult was from South America and studied part-time. A woman from Eastern Europe worked with the mental health lab. The diversity of the group of LHPs enabled a wider outreach and ensured that the work was not perceived as belonging to a specific interest group.

**Figure 1:**
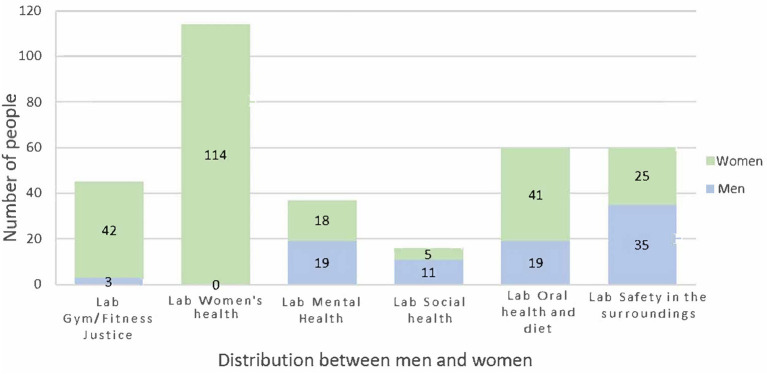
Participants by gender and lab.

**Figure 2:**
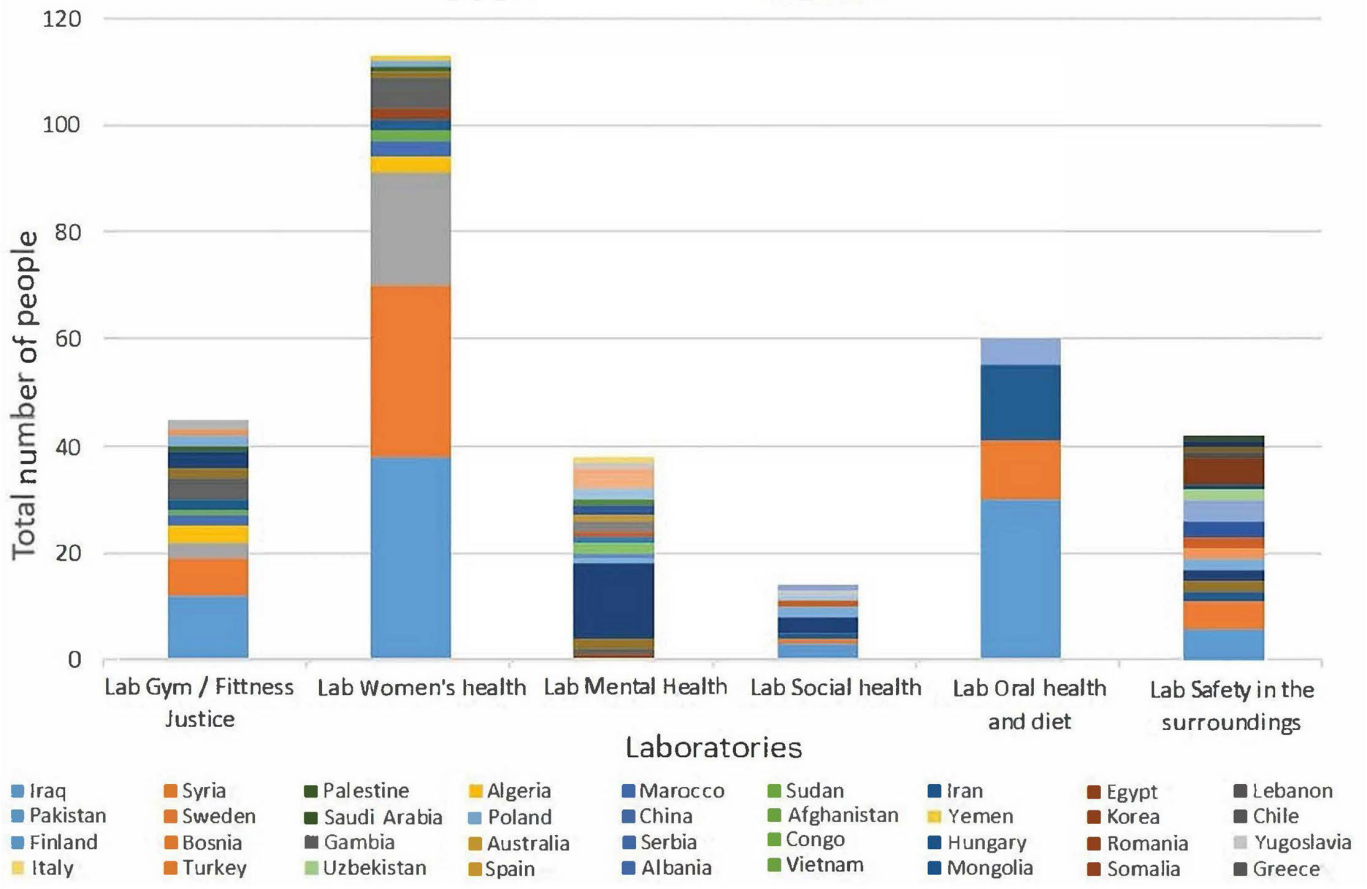
Participants by country of origin and lab.

The local stakeholders supporting the labs were representatives from social and primary care, primary school, non-governmental organisations, researchers from academia, sports associations, a private dentist, and a pharmacy (see Table 2). The academic partners were responsible for evaluating and writing about the programme and met with the LHPs during monthly HUB meetings and lab activities, as well as for planning and methodological support. Municipal partners contributed with staff in the local HUB and in the labs, as well as with administration and localities for meetings and activities. The LHPs and citizens together decided which partners they needed for the various activities, and the partnerships were evaluated every 6 months, together with the citizens. The different labs entailed different partnerships and collaborations. The LHPs mobilised 322 community members per week in the labs, of different ages and genders, and with different cultural backgrounds (see Figures 1, 2).

**Table 2:** Overview of actors involved in the six health promotion labs.

**Mental health**	**Women's health**	**Social health**	**Secure environments**	**Physical health**	**Oral health and nutrition**
**LHP 1**	**LHP 1**	**LHP 3**	**LHP 4**	**LHP 5**	**LHP 6**
Citizens with support from: Social workers Culture workers Primary care City care department IT company Red Cross Researchers/PhD students	Citizens with support from: Red Cross Primary care Health care City care department Sport associations Social work Employment agencies Researchers/PhD students	Citizens with support from: Red Cross Social care Culture workers Property owner FINSAM Researcher/PhD students	Children from school with support from: Schoolteachers Save the Children IT company Property owners Researcher	Citizens from the area with support from: Sport associations Social workers Culture workers Researchers/PhD student	Citizens from the area with support from: Social work Primary care Local pharmacy Oral health company Researcher/PhD student

*Each lab was led by a lay health promoter*.

Details concerning other aspects of the programme have been reported in publications concerning physical activity, safe environment for children, oral health and nutrition, and the overall health promotion programme (13, 17, 20–23).

### Procedure and Data Analysis

The Lindängen initiative has generated very extensive documentation throughout the course of the programme, and findings on various aspects have been reported in nine academic publications, as well as numerous reports to funders and the administrations. The present study on the role of the LHPs primarily focuses on the evaluations made in 2019, during the last stages of the period funded by the Swedish innovation agency Vinnova, including key conclusions by the partners and the LHPs themselves as documented and validated by participants, as well as their reflections on challenges for the continuation of the initiative beyond the period where Vinnova contributed to 50% of the budget. The study is based on multiple sources and research strategies (see Table 3 for an overview), including a) notes from regular monthly meetings with the LHPs in 2019 b) individual in-depth interviews with LHPs in 2019, based on a thematic guide and lasting for approximately 1 hour and c) three focus group interviews with local stakeholders working with the LHPs in 2019 (lasting 30–60 min), and one in 2020 after the period of external funding from Vinnova. The focus groups interviews were based on a guide for self-reflection and evaluation of CBPR partnerships developed by Wallerstein (19).

**Table 3:** Overview of documentation analysed in this study.

**Method**	**Material**	**Time**
Meeting notes in the local HUB	LHPs' discussions about the labs	Monthly meetings 2019
Individual in-depth interviews	Thematic guide—(focus on power mechanisms)	2019
Focus group meetings	Thematic Evaluation Focus group guide [focus on partnership and collaboration, see Wallerstein (11)]	Three meetings 2019 One meeting 2020

A qualitative content analysis by Elo and Kyngäs (26) was used to analyse all the data as a single unit of analysis. Transcripts were read to reach an overall understanding of the material, which was then condensed to meaning units corresponding to the aim and coded manually. Thereafter, the codes were interpreted and compared to find similarities and differences, and finally sorted into tentative themes by the authors.

### Ethical Approval

Informed consent and ethics committee approval were obtained at the outset of the programme (Reg. No. 2016/824 2018/591).

## Results

The following sections highlight the three themes that appeared in the analysis. Each of the three themes is discussed separately and with quotations from the interviews.

### Development of the Role and Function of the Lay Health Promoters—A Collaborative Process Situated in Local Places and Spaces

The labs and the lay health promoters (LHPs) had their activities scattered around the housing area. The choice of places for the activities and meetings was an important factor for the success of the Lindängen initiative, and for the work of the LHPs, in particular. These places were located within the neighbourhood close to people's homes and were therefore accessible to all in terms of time, effort, and cost required to get there. Many were familiar social spaces, created by the city of Malmö as inclusive communicative spaces for all citizens, regardless of age, gender, and ethnicity. Other social spaces had been created in housing areas where the property owners used the same criteria of inclusiveness. The people meeting there were empowered by the nature of these places to take more active and assertive roles in the processes. For the LHPs, these open spaces for communication were necessary to create an environment of inclusion. One of them explained:

*The prerequisite for mobilising was that there were open public places paid for by the municipality and property owners, where everyone was welcome and not only a specific group of citizens*. (LHP)

Once the themes for the co-creative labs had been set in the future workshops (16) and organised in a CBPR workshop for planning (2) together with all stakeholders, the specific content of the labs was elaborated and continuously re-evaluated directly with participants in the activities, and with other inhabitants. Similarly, the forms, roles, contributions, and responsibilities of the various stakeholders were also reconsidered and elaborated in the course of the processes, to meet the challenges and needs that emerged. A representative from Save the Children Sweden expressed the importance of the LHPs in that process:

*In a neighbourhood like this, processes that create trust are needed. The (lay) health promoters know their immediate surroundings and can create trust among the citizens. They become the link that is needed between citizens and society to create trust. (Save the Children)*

In driving and coordinating the activities, the LHPs had to find forms of work to manage the wide range of functions and responsibilities they had within the programme. In their position as brokers—mediating between actors from formal institutions and the inhabitants and participants in lab activities—the LHPs had to define their own stances. The main challenges in the early stages had to do with building self-confidence, coping with stress, managing practicalities, or dealing with group dynamics during activities and meetings with participants in the co-creative labs. The CBPR approach and collaboration with academic actors were crucial in enabling the LHPs to work with such challenges. Over time, however, as the LHPs gained confidence and experience, the inherent tensions between the interests of the inhabitants and the structures and expectations of institutional stakeholders became a central issue. Two dimensions of the processes appeared to be particularly important: the time and personal involvement required to mobilise inhabitants and gain credibility, and the use of a CBPR approach including academic participants. This supported the processes and led to mutual learning.

### Credibility and Mobilisation for Democratic Processes

At the outset, the LHPs used their personal networks in the neighbourhood to gradually expand citizen involvement in the labs. They also paid careful attention to the participation process as one of them explained:

*I think it has become more democratic; the participants have to decide themselves (in the labs), so most of them learn how a democratic system works*. (LHP)

At every stage trust was the most important factor. Trust in the LHPs and in the benefits of the activities was gradually built up through experiences from the activities, discussions, and the consistent commitment of the LHPs to work with the issues people felt were relevant to their lives and needs.

The fact that the organisation and the interventions corresponded to the needs and themes that residents had expressed during the early workshops was also an important factor in building trust. The engagement in the future workshops and the CBPR planning empowered the citizens, so that they felt ownership over the process in building up the programme. One of the LHPs explained that working from a community base was a novel structure for them:

*When we were employed before, we were forced to work from within these organisational structures and needs—here work is more based on the wishes and needs of the people. Yes, it is citizens in the community who are building the structure*. (LHP)

While public institutions are under pressure to measure success in terms of quantity of services delivered, in the Lindängen programme, the quality of interaction was paramount, as was the relevance according to people's own perceptions, rather than external criteria. This was a question of listening and giving space for residents to voice their thoughts, rather than of spreading “information” to raise awareness. Letting those processes take time thus became the foundation for open dialogue based on mutual trust, also on the part of the local stakeholders. As one of the representatives from the local social services explained:

*During the project, the (lay) health promoters have grown together. It is like a process of trust where the shared goal has been that together we are able to make change happen*. (Malmö City)

Throughout the course of the programme, mutual trust among partners and from the community was sustained and deepened by a better understanding of each other's perspectives, as well as insights into the constraints under which the partners operate. Above all, trust was maintained by seeing competence and commitment in action, and by experiencing tangible benefits.

### Use of a CBPR Approach Can Change Power Relations

The LHPs were able to develop the model for how to organise their work and mobilise residents because they themselves came from the neighbourhood and were part of the communities they served. This had more to do with who the LHPs were as individuals and how their personal life stories resonated with the community, than to which ethnic group they belonged. People coming from outside the neighbourhood would not have had the necessary knowledge or competence to achieve this, nor would residents have felt that they knew enough about outsiders' background and character to entirely trust them. The central position that the LHPs gained through the programme meant that they also had to be careful about how they managed this power in the labs. As one of them reflected:

*We know that we are an authority in the labs, but we have something that governs us as well. We keep a certain direction (based on the CBPR planning). For example, I can*'*t control the activities and create (an activity) that the citizens don't want to come to*. (LHP)

The process of building relationships and mobilising inhabitants for activities in the labs involved close person-to-person interaction. Several of the labs concerned personal and emotional issues regarding mental health and family situations.

Both the personal nature of the interaction, and their sense of responsibility with regard to serving the community, meant that besides organising activities in the co-creative labs, the LHPs spent a considerable amount of time providing services to the residents of the neighbourhood, helping with practical issues as well as offering emotional support. Such support had to meet the times and circumstances when people actually needed assistance, rather than following time schedules and formats set in advance. One of the issues the LHPs faced was being overstretched and maintaining a balance between their own needs and the responsibility of serving the community.

Through their involvement in the research process and in the collaboration with the other stakeholders, the LHPs were able to support a process of learning among the other actors. The PhD students and researchers from academia particularly benefitted from the LHPs' knowledge about the context, learning about the perspectives of the residents and the situations that people in the neighbourhood experience. This insight was important to understand sustainability:

*Sustainability depends on the citizens*' *power…The (lay) health promoters are central in building a sustainable society*. (Researcher from the university)

It was not easy for the LHPs to manage their position as brokers, being part of separate contexts and negotiations that followed different kinds of logic. However, the fact that they were a group of several individuals experiencing the same tensions made it easier for them to support each other during the processes. They could reflect jointly on what was happening and set up strategies to deal with the situation. The LHPs were also able to support each other with the conflicts and tensions that emerged during activities with the citizens, or in empowering the women in the community to take control over decisions that had to be made in the labs.

*I think it became understandable what democracy is during the process in the lab. The participants are used to authoritarian leaders, but I tried all the time…No! we have to decide for ourselves*. (LHP)

The participants also experienced empowerment processes from their practical learning in the programme. One of the LHPs explained:

*But what I notice... we have been educating each other through the work and with all this knowledge comes very much a power shift. I would say, it teaches us that knowledge is power for all citizens*. (LHP)

Knowledge relating to cultural and highly contextual codes was articulated towards external stakeholders and further developed through joint reflection. Importantly, the orientation and framing of this development, the meaning that is given to situations or events, and the decisions about what is relevant, were driven by the LHPs. This was defined, both based on discussions during their meetings with the other LHPs, and by drawing on the response and ideas that emerged among participants in the activities. Continuous dialogue with the academic partners, input from experts on specific issues, and formal training in participatory research methods, all enabled the LHPs to assume this key function in the collective research processes related to power mechanisms in the labs. As one of them explained:

*We have a great position of power as (lay) health promoters. It's important that we don't use (our position) to force citizens to do something they don't want. That works only one or two times, the third time they won't do the activity—and then everyone turns their backs on us. We create this together*. (LHP)

The LHPs' participatory work also started a discussion within the non-governmental organisations about empowering citizens through a bottom-up process:

*The work of the (lay) health promoters is starting to make a difference at the policy level, now it is through them, that we can show in the organisations that this is how we should be working from now on*. (Red Cross)

## Discussion

### Empowerment Through Action and Drawing on Tacit Knowledge

The processes during the Lindängen programme can be understood in terms of empowerment (27). While the notion of empowerment can be contested and may even in certain contexts be used to shift costs and responsibilities towards disenfranchised groups, within the framework of CBPR there is an emphasis on fostering an emancipatory stance. Wallerstein defines empowerment in the context of health equity as: “[a] *social action process that promotes participation of people, organizations, and communities towards the goals of increased individual and community control, political efficacy, improved quality of community life, and social justice*” [(28); p. 198]. At the individual level, Zimmerman's (29) definition of psychological empowerment includes people's perceived control of their lives, sense of community, level of participation in community change, as well as critical awareness of socio-economic or political contexts and targets for change.

The LHPs in Lindängen played a key role in the collaborative model for cross-sectoral work and community engagement. This role went far beyond simply being employed to perform functions and implement policies defined by a central organisation or public institution. In theories of situated learning (30), knowledge is embedded in situated and embodied practices, in the communities where the practices take place. From this perspective, brokers who are part of different communities of practice mediate, not only between cultures, but also between the contextual and tacit dimensions of practice that take place in the various communities. This understanding of knowledge as carried by individuals, practices, groups and specific contexts, contrasts with formalised knowledge systems, which see knowledge as neutral and explicit parcels of “information” that can be readily transferred across contexts. Working in the programme, the LHPs learned that all this tacit knowledge (31) was also an important part of their own power and could be useful to empower the community. As Kane et al. (32) conclude, for community health workers to be able to empower the citizens and communities they serve, it is essential that they themselves be and feel empowered.

For the other stakeholders and academic participants, the most important knowledge transfer that took place during the programme, was from the community, changing their mindset and preconceived ideas about so-called “marginalised groups” or low-income neighbourhoods. Since the academic participants were active in different professional education programmes, they transferred this knowledge back into the education system.

### Institutional Constraints on the Role of Community Health Brokers

The function of mediating “brokers” is not unique to this project (12). Both NGOs and the social sector occasionally employ brokers from “at-risk” groups or individuals who live in vulnerable neighbourhoods [see e.g., (12, 19, 33)]. Although such institutionalisation ensures funding and brings the benefit of greater continuity, brokers tend to have little autonomy. They are “stuck in the systems” they work for, and their work is limited by the constraints of the employing organisation or authority. Such structures leave little scope for community empowerment and do not contribute to alleviating silo effects between sectors (34). Thus, Rodrigues Fausto et al. (35) observe that in Brazil, the integration of community health workers into the institutional health system has confined them to a more technical role and limited their political role.

In countries like Brazil or the US, local communities are sometimes strong enough to employ health brokers themselves. While health workers funded by community-based organisations can work holistically, address social determinants of health, and engage in advocacy, by contrast, health workers employed by institutions tend to deliver specific services consistent with the responsibilities and priorities of their employers (36, 37). Torres et al. (36) thus note that tensions between community needs and the structures of institutions have been observed in Brazil, Iran and the US. The issue of how community health workers balance their roles in community action, advocacy, and intervention has also been discussed by Rosenthal et al. (38) in the US context. Anderson and Cidro (39) point to examples of tensions experienced by indigenous academic women researchers doing CBPR in their communities. Henderson and Kendall (40) emphasise the significance of addressing broader issues that people consider important, seeing this as the foundation for the credibility of community health workers. Social exclusion and disempowerment related to status, class, or perceived ethnicity, have negative impacts on health and well-being (41), while the importance of addressing the root causes of social inequities is also highlighted in the WHO Health 2020 framework for Europe (42).

### Community Action in the Swedish Context

In Sweden, neighbourhoods and groups are seldom organised into strong communities as in Brazil or the US. Besides carrying on the work with community engagement and developing the model together with other stakeholders, the LHPs in Lindängen analysed the constraints of the current system, and organised to create the structures they need to continue and further develop their work for the community autonomously, relying on the community itself rather than on external support. In Sweden, to function in the public space and have an impact, any collective activity often needs to be formalised in the form of an association (43). The LHPs in Lindängen have therefore formed an association, so that they can be employed and continue to perform services for the community, but at the same time be free from the silo structures of the various sectors of the public institutions. In Canada, culture brokers, health brokers, “animators”, and other mediators have been employed by the public authorities to perform similar functions, but generally from the perspective of implementing top-down policies (44). In Lindängen, the approach has been closer to advocacy for the community, shaping spaces where citizens' perspectives can be discussed, both within the neighbourhood and with the larger actors of the initiative.

A crucial observation made by Torres et al. (36) is that funders are willing to finance service delivery, but rarely invest in community development or in addressing social determinants of health. Work for the community will therefore tend to take place on the health brokers' own time. Torres et al. (36) noted that most health brokers worked part-time and had to take other jobs to make a living. Similar issues could be observed in the Lindängen initiative. The partners were happy to provide support or make joint applications for external funding, and long-term funding has been secured for several aspects of the organisation. However, several partners from healthcare and the city have been reluctant to offer regular employment to the LHPs. In the Lindängen case, this has partly been a matter of institutional constraints that required specific qualifications for particular positions, or the inability to find a suitable rubric in budgets for this kind of work. An additional concern has been the protection offered by Swedish labour law (LAS) to permanent employees, with restrictions to temporary contracts in the longer term. This is why employers tend to prefer short-term projects. Such difficulties in negotiating continued commitment from certain partners has partly undermined the process of trust building established during the programme. As of December 2021, two LHPs have been employed by NGOs on a long-term basis with funding from the city and local property owners. Other LHPs are funded by short-term projects.

### Potentials for Cross-Sector Collaboration and Structural Limits

The Lindängen programme was intended to function across sectors and allow collaboration between a wide range of stakeholders, to avoid the silo structures that public institutions suffer from. In the programme, funding came through the overall project, which gave some scope to work for the community in innovative ways across institutional boundaries. However, the project did not result in a joint commitment from participating stakeholders to share the cost for employing all the LHPs permanently. At the same time, if the LHPs were to be employed by individual institutions or stakeholders that were not committed to the HUB and its engagement to work in a CBPR manner, this would considerably restrict the ability of the LHPs to work autonomously across sectors and to continue to engage in developing community capacity. Similar conflicts in loyalty have been observed in several neighbourhood development initiatives in Sweden (4–7). Throughout the programme, the LHPs did not only function as culture brokers, relaying different perspectives on health and health promotion between residents of the neighbourhood and the institutional actors. Rather, the LHPs played a central role in determining the priorities and structure of the project from the outset, mobilising and training the community developing and conducting activities, evaluating progress, and developing new priorities and forms of action based on this experience.

### The Significance of Funding Structure and Temporal Constraints of the Project Form

Even if the city of Malmö were willing to employ the LHPs as employees of any of the existing institutions or organisations, the LHPs would be bound by the priorities and hierarchies of that organisation, as well as by the rules and regulations that define the modalities and scope of its work. In the best of cases, the vision of local authorities would be in line with the local communities, but authorities' autonomy is nevertheless restricted to finding locally appropriate ways of implementing centrally defined policy goals. While the NGOs have somewhat more freedom in defining their field of action, they are equally bound by funding constraints. Reliance on external funding tends to fragment the scope of each “mini-project” and to limit its duration. Many of the people working in these organisations are engaged voluntarily or for short periods, while the permanent staff tend to have administrative or fundraising roles. The larger NGOs may benefit from more continuity in funding, but tend to organise priorities centrally, and often have strict hierarchical organisations, similar to public institutions. Private sector actors within the programme had more flexibility in this respect, as well as being less dependent on external funding for continuing the work in the form of projects (13). Thus, the housing companies in Malmö were involved in an initiative to set up a foundation which partially funds social programmes for children and families that support LHPs.

### Formal and Informal Perspectives on Work and Time

Torres et al. (36) highlight four important dimensions in the work of the Multicultural Health Brokers co-operative (MCHB Co-op) in Edmonton Canada: (a) articulating, reflecting on, and monitoring the health brokers' practice (b) enhancing the capacity of the organisation (c) developing a market niche and (d) seeking intersectoral cross-governmental collaboration. By comparison, the LHPs in Lindängen expressed the strain of having to work much more than the time formally allotted for their involvement. This had to do partly with the complexity of their role which, like the MCHB Co-op (36), was highly challenging, but was also linked to their close connexion with the inhabitants and the limit to their scope of action. An issue observed by members of the community health cooperative investigated by Torres et al. (36) was that the collective decision-making process was time-consuming. Furthermore, health brokers in Edmonton had to work voluntarily beyond the recognised hours of service to meet the needs of the families. The issue of invisible non-recognised work was also noticeable for the LHPs in the Lindängen programme. These experiences expose a tension between recognised work—corresponding to external perceptions—and the actual use of time necessary to work closely with a population in a participatory manner. It also points to the more general problem of voluntary work and work in the caring professions: individuals feel bound to do their utmost to serve a community or people who need their care and can be exposed to burnout or conflicts with their personal lives. Underfunded organisations and institutions thus tend to exploit the engagement of health workers, perpetuating a situation where the burden and responsibility is placed on individuals working directly with people needing their support, rather than carried through adequately resourced structures.

### Main Strengths of the Programme Design

The central position occupied by the LHPs was a key element for the success of the programme as a whole. Combined with a design that was open to innovation and with a strong focus on actively engaging members of the community, the initiative has resulted in empowerment and a new association, LindängenKraft (LindängenPower), driven by the community and the LHPs, with the assistance and help from the HUB's organisations on the neighbourhood level. The decision to create this association at the end of the main period of external funding is a strong indication that the neighbourhood residents experienced benefits and wish to support the continuation. It is still too early to assess how effective the association will be.

Based on their experiences in the programme, the LHPs gained clearer insights into the institutional and structural conditions that impact their community (13). The position they had in the process allowed them to take political and social stances towards various issues and to start to concretely address the social determinants of health in the community, as well as expressing recommendations to policymakers. A significant feature of the initiative was also that the LHPs were supported by participatory action researchers from the university. The support enabled the LHPs to be more effective in creating the model for the initiative, and the processes were integrated with participatory action research (PAR) from the outset. The LHPs are currently taking courses on PAR methodologies at adult education institutions and popular education organisations, and use these methodologies in the health labs that they run.

One of the reasons why the LHPs' methods of working were readily accepted by local authorities in Malmö—and a reason to hope that the project of an independent association will succeed—may be the strong popular education tradition on which Sweden's welfare system was based historically. In the early 20th century, workers and their organisations initiated so-called study circles on different topics, allowing people to continue their education. Later this form of collaborative learning was spread to the industry, where labour unions participated in organisational changes through action research in the study circle form. As the welfare state now shrinks and top-down approaches are proving inadequate to meet the needs of the population, we may well be witnessing a situation where popular education at grassroots levels (45) could play an important role in addressing the social determinants of health. This orientation for health promotion is consistent with the ethos and ambitions of community empowerment outlined in the Ottowa Charter (46) and the Jakarta Declaration (1) for health promotion. The experiences of the LHPs in the Lindängen programme thus offer valuable insights into both the opportunities and the constraints for such work in Sweden today.

Studies that have been carried out on various outcomes of the Lindängen initiative (13, 17, 20–23) indicate that it has in many respects been successful. However, the present study also points to important structural issues in collaboration between public institutions and communities. Similar problems have been observed in Swedish studies on neighbourhood development (4–7) and in the international literature on cultural brokers in community health work. The present study thus contributes to the literature on these issues by providing a recent Swedish example of attempts to achieve collaboration in the context of community health promotion.

### Limitations and Recommendations

Among the main conclusions that can be drawn from the programme is the difficulty in creating permanent positions for the function of LHP, because this function does not fit into the structural and legal constraints under which institutions work. Participants in the Lindängen initiative believe that meaningful work cannot be conducted if the process is not driven locally and allowed to take time. Having lived through the processes and collectively reflected on the positive outcomes, as well as the obstacles faced during the programme period, the LHPs do not want the work to be deviated and channelled back towards institutional priorities. Instead, they feel it is vital to continue the processes of empowerment for themselves and for the community. A crucial point for similar programmes is therefore how LHPs are funded. Employment by institutional actors entails constraints that would substantially hamper the LHPs in their roles, while not-for-profit organisations are comparatively weak and dependent on external project funding.

Another important lesson from the Lindängen programme is that LHPs cannot simply be recruited from the population and employed within health or social services. First, they need to be accepted as individuals by the residents they are serving. Second, for the LHPs to invest in the processes and continuously develop the structures beyond a set of initial tasks or responsibilities, the aims of the initiative have to be in alignment with their personal values and convictions. To mobilise the local population and maintain credibility, their loyalties must be clearly on the side of the community. Finally, the programme needs to offer sufficient resources and freedom of action to engage in activities that actually benefit the community, in line with the priorities expressed by the inhabitants. These activities need to be continuously reassessed concerning feasibility and responses from the community, rather than being primarily defined by accountability towards external institutional structures and constraints.

### Directions for Future Research

The new association created by the LHPs at Lindängen mobilises local resources, and benefits from credibility with respect to both community and other stakeholders achieved in the course of the programme. The form of association offers several promising opportunities in a Swedish context, since association members can freely define their agendas and priorities. However, associations in Sweden also suffer from many of the general vulnerabilities of the not-for-profit sector. Due to a unique design, the community health development work conducted by the LHPs in the Lindängen programme provided them with a key role in developing content and priorities based on community input, as well as participating in coordinating and steering meetings with stakeholders at all levels. The programme design has also offered multiple opportunities for collective reflection, as well as training in participatory research methodology. An interesting avenue for future research would therefore be to follow the work of the new association founded by the LHPs and see whether these tools and experience can help them overcome typical challenges described in the literature in relation to community health promotion.

## Data Availability Statement

The datasets presented in this article are not readily available because data primarily consist of interviews, meeting minutes and field notes. More stringent restrictions apply since participants belong to vulnerable groups. With such a small number of interviewees, it would be possible to identify participants even with anonymization. Requests to access the datasets should be directed to margareta.ramgard@mau.se.

## Ethics Statement

The studies involving human participants were reviewed and approved by Regionala etikprövningsnämnden i Lund (Regional Ethics Committee of Lund). The participants provided their written informed consent to participate in this study. Written informed consent was obtained from the individual(s) for the publication of any potentially identifiable images or data included in this article.

## Author Contributions

MR was PI for the overall programme and performed the interviews. HA and MR participated in the design of the study and the analysis. HA wrote the initial version and then discussed and developed it further together with MR. Both authors have read and approved the final version of the manuscript.

## Funding

The current work was part of a larger project funded by the Swedish Innovation Agency Vinnova with the sum of SEK 8,6 m (DNR 2016–00421, 2017–01272), and the Faculty of Health and Society, Malmö University.

## Conflict of Interest

The authors declare that the research was conducted in the absence of any commercial or financial relationships that could be construed as a potential conflict of interest.

## Publisher's Note

All claims expressed in this article are solely those of the authors and do not necessarily represent those of their affiliated organizations, or those of the publisher, the editors and the reviewers. Any product that may be evaluated in this article, or claim that may be made by its manufacturer, is not guaranteed or endorsed by the publisher.
